# Benefits of an educational program for journalists on media coverage of HIV/AIDS in developing countries

**DOI:** 10.1186/1758-2652-11-2

**Published:** 2008-09-22

**Authors:** Jorge L Martinez-Cajas, Cédric F Invernizzi, Michel Ntemgwa, Susan M Schader, Mark A Wainberg

**Affiliations:** 1 McGill University AIDS Centre, Lady Davis Institute for Medical Research, Jewish General Hospital, Montreal, Quebec, Canada

## Abstract

**Objective:**

a) To assess the suitability of the curriculum content and didactical quality of information delivered to educate journalists in the J2J program in HIV/AIDS (process evaluation) and b) to explore the effects of such programs on journalists' reporting of HIV/AIDS related information (outcome evaluation).

**Design:**

Descriptive study.

**Methods:**

For the process evaluation, each J2J program curriculum was evaluated for accuracy and pertinence by individuals with high familiarity with HIV/AIDS research. For the outcome evaluation, a survey of J2J attendees and evaluations of the program lectures by attendees were performed in chronological order to determine their perception on usefulness of the program.

**Results:**

Overall, the J2J curriculum is successful in providing journalists with a clear understanding of the current HIV/AIDS medical research objectives and issues with most journalists reporting an increased ability to better investigate and disseminate accurate information on this subject. Furthermore, the journalists surveyed reported positive community responses directly as a result of the J2J training.

**Conclusion:**

The J2J program helps to increase global awareness of pertinent HIV/AIDS concepts. Through this professional development strategy, journalists from around the world may help to amplify efforts to prevent new HIV infections and quench the dissemination of inaccurate information and folklore.

## Introduction

The detrimental impact of the acquired immunodeficiency syndrome (AIDS) on global health has continued since the first reported cases of human immunodeficiency virus (HIV) infection in the early 1980s. Thus, facilitating worldwide awareness of HIV/AIDS is of paramount importance in public health campaigns aimed at prevention of new infections.

The dissemination of HIV information is a task largely undertaken by community health care workers, advocacy groups, and journalists. Of these professions, journalists probably are the most able to efficiently disseminate pertinent information on a global scale [1,2] and must do so in languages that are understood by the general public. By contrast, misinformation about HIV/AIDS might result in an increase in HIV transmission.

Thus, effective communication between HIV/AIDS research groups and journalists from around the world is essential if we are to improve the understanding of HIV/AIDS worldwide. This was the premise that led to establishment of a Journalist-to-Journalist (J2J) HIV/AIDS training program as a component of the International AIDS Conference in 2002.

The program was developed as a satellite meeting by the National Press Foundation (NPF) in advance of the main conference, the purpose of which was, "preparing selected journalists to cover the International AIDS Conferences, and then to continue to cover the subject at a higher level than previously imagined." It is important to note that the journalists accepted into the program did not have specialized scientific training.

The program was first launched at the Barcelona International AIDS Conference in 2002, and has been held three other times since then in Bangkok in 2004, Toronto in 2006, and Sydney in 2007. Fellows are invited to participate based on their journalistic competence and experience after submitting a successful application to attend. Preference is given to journalists from developing countries since such areas are considered to be most at risk for new HIV infections and because journalists from developing countries are often least able to afford the costs involved in participating in such a conference.

The invited individuals had to be journalists or communicators in any field, had to have previously written or broadcast about HIV/AIDS and have to had the support of their supervisors to attend. They also needed had to supply a printed or video version of a piece that they had done in the field of HIV/AIDS. After these criteria had been met, a second evaluation involved ability to speak English, the type of medium used the candidate and the country of origin so that as many countries or regions as possible would be represented. Financial assistance for travel, lodging, registration, and meals for the duration of the J2J program and conference was provided by the program that is funded by a grant to the NFP by the Bill and Melinda Gates Foundation.

Thirty nine of 74 journalists who were invited attended the Barcelona J2J program, but this number dropped to only 9 of 75 for the Bangkok conference primarily because poor communications from the conference organizers to members of the journalistic community. In contrast, 95 of 105 invited journalists attended the Toronto conference and 42 of 44 invited journalists attended the Sydney program.

Researchers in each of the basic, social and clinical sciences strongly agree with the crucial role that journalists can play by accurately informing the public on issues that relate to the global HIV/AIDS epidemic [3-5]. Prevention of HIV infection, accessible health care for HIV positive individuals, and public policy are all issues that may be highlighted through journalism. Furthermore, journalists are often able to translate the objectives of HIV/AIDS advocacy and research groups into language that is more likely to be understood by the communities to which these messages are targeted. In fact, programs on HIV prevention, stigma, the health care needs of those infected by HIV/AIDS, and advocating for government intervention can all be directly affected by what journalists choose to report.

### Purpose of this evaluation

The authors of this report (two Ph.D.s, one M.D., and two Ph.D. candidates), all very familiar with the HIV/AIDS scientific literature, were asked to evaluate the J2J program in order to:

a) assess the relevance of the curriculum content and didactic quality of information delivered to journalists (process evaluation) and,

b) explore the effects of such programs on reporting of HIV/AIDS related information (outcome evaluation).

Our secondary objectives were to

c) assess journalists' perceptions as to how this training program affected their coverage of HIV/AIDS, and

d) determine whether the program had resulted in improved provision of information to communities about truths and misconceptions about HIV/AIDS.

### Methods used for assessment

Our team was provided by the J2J program organizers with the following material for evaluation of the program:

a. E-mail addresses of all participant journalist fellows who possessed such an address.

b. A large sample of news stories on HIV/AIDS written by journalists who attended the training sessions and conferences.

c. Evaluation reports of the Bangkok and Barcelona programs previously prepared by the National Press Foundation

d. Evaluations by journalists Sydney program.

e. Data accessible online from a number of slide presentations delivered in each of the following J2J training programs (Barcelona 2002, Bangkok 2004, Toronto 2006, Sydney 2007). Presentations from Sydney also included voice recordings of scientific presentations.

Careful study of the J2J curriculum (included as part of each conference program) was completed by at least two evaluators. Each evaluator issued a descriptive statement on the completeness of the program by answering the following questions:

Is the content of the J2J curriculum suitable and complete?

What key subjects were lacking?

What subjects might be excluded?

After each individual evaluation, a group discussion resulted in agreement on the completeness of the curricula. In the same fashion, a sample of 24 slide presentations (available online) were evaluated for relevance, complexity, organization and quality of slide presentation. Each slide presentation was scored using the following scale: 1 = poor, 2 = fair, 3 = good, 4 = excellent.

In addition, journalists' evaluations of the Sydney conference J2J program (n = 42), which had used the same scoring scale, was taken into consideration.

To establish the benefits of the program, two types of analyses were performed. First, a random sample of 39 news reports (of 84 available in English or with an accompanying English translation) completed by journalists who participated in any of the J2J programs was examined for relevance and accuracy (using the scoring scale described above). These 39 news reports represent a sample of 46% of the total of reports available in English. Each report was reviewed by at least two members of our team. In cases of non-agreement, which were very rare, the senior author of this paper made a definitive assignment of grade.

A short survey in the form of a questionnaire (Additional file 1) was also distributed to all participating journalists to assess the overall perceived benefits (if any) of the J2J program. Journalists' responses were compiled, reviewed and analyzed.

## Results

### 1. Evaluations of curricula

For the Barcelona and Bangkok conferences, comments from attending journalists were available in reports prepared by the NPF [6,7]. The Barcelona, Bangkok and Toronto conferences were each multidisciplinary and the J2J programs at those conferences were intended to enable journalists to acquire necessary knowledge of a meeting with a broad scientific, social and cultural agenda. In contrast, the Sydney conference focused on biomedical research, improved treatment, and prevention strategies, as well as on obstacles toward attainment of these goals. The content of each J2J program is presented in Table 1.

**Table 1:** Curriculum of each J2J program at the International AIDS Conferences

Conferences with interdisciplinary focus			Conference with biomedical focus
**BARCELONA**	**BANGKOK**	**TORONTO**	**SYDNEY**

**Basic and clinical science**	**Basic and clinical science**	**Basic and clinical science**	**Basic and clinical science**

Basic Science of HIV/AIDS	What HIV Does in the Body	HIV/AIDS & Vaccine Research	Living With HIV/AIDS
What HIV/AIDS Does in the Body	Treatments, Current & Future		All You Need to Know About Microbicides
Treatments, Current & Future			PLENARY PREVIEW: T-cell loss, immune activation and potential therapeutic interventions
			PLENARY PREVIEW: Understanding the Task: ARV Rollout and research issues in the developing world

**Medical and therapeutic issues in HIV**	**Medical and therapeutic issues in HIV**	**Medical and therapeutic issues in HIV**	**Medical and therapeutic issues in HIV**

Prevention	Tracking HIV/AIDS: Numbers that Count: The Demographic and Health Surveys (DHS) project provides quality data on the What, Why, Where and When of HIV/AIDS	Epidemiology 101	PLENARY PREVIEW: Pediatric Therapeutic Issues
Access to Treatments	Preventing HIV/AIDS	Developing HIV Prevention Options for Women: Microbicides	
	Integration Of Prevention Into Treatment Programs And Other Issues Posed By Treatment Access	Female Condoms	
		Paediatric AIDS	
		HIV/AIDS and nutrition in rural areas	
		HIV/AIDS in Latin America and the Caribbean, Asia and Africa: The differences between the epidemics, the different responses, and the different issues in various regions	
		HIV/AIDS & TB	

**Journalism and HIV/AIDS**	**Journalism and HIV/AIDS**	**Journalism and HIV/AIDS**	**Journalism and HIV/AIDS**

Journalists' Discussion Groups + session	Beyond He Said/She Said: Giving Depth to HIV Stories	Discussions on Covering HIV/AIDS	Plenary Preview: Male Circumcision
Journalists' Discussion Groups + session leaders	Field Trip: Presentation: AIDS in Thailand	Special Presentation Ontario Room The Blood of Yingzhou District	*Journalist to Journalist Discussion*: AIDS Denialism What it is, how to recognize it, how to dispute it, with a focus on a recent Australian legal case
Practical Tips & Story Ideas for Covering the XIVth International AIDS Conference	Journalists' Discussion: Privacy, Reporting & HIV/AIDS	Congratulations and a Charge to Journalists	*Journalist to Journalist Discussion*: The Multiple Layers of AIDS Coverage
Tracking the Money	Trends, Trends & Q&A	Tips for covering the Toronto conference	Tips for Covering the Sydney Conference *Overview of different tracks from the conference: what they mean, what they'll cover, how to choose what to attend*
News & Numbers	Training the Trainer	HIV/AIDS in Context	
	Health Beyond HIV/AIDS & Why the Media Should Care	Looking Beyond Toronto to Mexico City in 2008	
		Trends and Q&A	

**Social and economical sciences**	**Social and economical sciences**	**Social and economical sciences**	**Social and economical sciences**

Economic & Medical Consequences of the Epidemic	Macroeconomics & AIDS	The Stigma Faced by People Living With HIV/AIDS	A New Initiative on MSM
Myths & Misperceptions	AIDS Orphans & Vulnerable Children	Human Rights & HIV/AIDS	Sex Workers: Part of the Solution, Not Part of the Problem
	AIDS in Context		

#### Curriculum Completeness

We observed a progression in the quality of the curriculum throughout the J2J series from the initial program attempt in Barcelona. The J2J program content was adjusted based on feedback from journalists after each J2J event. This was done both with respect to content and the topics for lectures at the J2J satellite meeting. A succinct assessment of the content of each J2J program follows:

#### Barcelona 2002 J2J program

This program was graded as fairly complete by our team of evaluators. The agenda allowed ample time for discussion and interactive sharing of ideas between experts and attendees. It included three lectures that introduced scientific and biomedical concepts and terminology frequently used in HIV/AIDS research. The Barcelona program also included discussions of economic and cultural issues surrounding HIV/AIDS. However, it was pointed out that the program would have been strengthened if a visit to local HIV care facilities or with community-based HIV/AIDS health groups had been arranged. It was also felt that socioeconomic issues surrounding the pandemic needed more attention.

Our evaluation also revealed that the Barcelona J2J program did not contain adequate information on how decisions are reached regarding the efficacy of drug interventions. This was in spite of the fact that the intention was to enable journalists to recognize basic principles of good scientific methods, especially in therapeutics and efforts to prevent transmission of HIV.

Journalists need to have basic tools to be able to identify overtly false science, which can be a common and widespread cause of public misinformation. Also lacking was an introduction to epidemiologic terminology frequently used to address public health issues.

#### Bangkok 2004 J2J program

Compared to the Barcelona J2J curriculum, the reviewers perceived the Bangkok J2J program as more complete. Of note, the reviewers found that a session on issues of people living with HIV (PLWHIVs) adequately allocated time for journalists to become informed of the diverse needs of PLWHIVs, including the issue of HIV-related stigma. Journalists who attended this series of seminars acknowledged the opportunity to speak to HIV/AIDS activists.

Topics that were determined to be insufficiently represented at this J2J satellite included:

1) An introduction to principles of scientific research.

2) Development of tools that enable journalists to ask the right questions about epidemiologic research.

3) Discussion of how the needs of PLWHIVs might be met by local government and non-government organizations.

Also lacking was a specialized seminar on how to access HIV/AIDS data on prevalence, trends, projections, public programs, as well as obstacles toward implementing HIV/AIDS health programs in various countries.

It was pointed out that assessments of local health agencies, government and non-governmental organizations (NGOs) would have been beneficial.

Some of the Bangkok J2J delegates felt that the biomedical research lectures presented at the J2J Satellite were burdened with excessive detail. There were also requests for implementation of country-specific J2J professional development curricula.

#### Toronto 2006 J2J program

The overall J2J satellite offered a rich epidemiological and cultural experience. On the other hand, the Toronto J2J curriculum was felt to be lacking in seminars about clinical research methodology and on how to decipher scientific jargon commonly used among HIV/AIDS scientists. Presentations on HIV prevention were appreciated by the delegates as were lectures on behavioral and medical interventions.

#### Sydney 2007 J2J program

The Sydney IAS 2007 conference focused primarily on biomedical research and the J2J program prepared for this through a comprehensive curriculum that spanned several days before the conference. The J2J organizers also offered comprehensive discussions on particular issues that were anticipated to be especially important. An introduction to scientific jargon was presented in the context of a session on vaccines and microbicides. A more general introduction to scientific jargon might also have proved useful. The reviewers felt that an informative session describing how certain scientific results are chosen for presentation at international conferences should have been included and also a session on how decisions are made by individual scientists to present their work.

In all J2J programs a paucity of participants from the private sector was evident. This is despite the fact that the drug companies are well represented at every IAS conference. It therefore seemed strange that this sector was not better represented in the J2J program.

A post-conference follow-up meeting was absent from the program. Such a meeting would serve to reinforce understanding of key issues/topics and enable journalists to clarify what they have or have not understood.

### 2. Journalists' Evaluations of J2J Sessions

At the Sydney conference, we gained access to journalists' evaluations of each presentation in the context of the J2J program. On average, journalists gave grades of excellent or good to fourteen of the sixteen presentations delivered. Thirteen of the sixteen lecturers (76%) were evaluated by attendees as good or excellent. The average grade for all lecturers was 3.08 which was comparable to previous averages from Barcelona and Bangkok, i.e. 3.1 and 3.19, respectively (maximum score is 4.0). Overall evaluations by journalists were good or excellent for each topic covered. Only three of thirteen presentations failed to score in the excellent range.

### 3. Assessments of presentations by the evaluation committee

Our committee evaluated 24 J2J presentations available online on the basis of relevance, complexity, organization, slide quality and background information. Only two presentations had an average score less than 3. The area in which presentations were frequently weak was in slide quality (five of twenty-four had poor quality and ten had fair quality). The content of all presentations, except two, was considered to be highly relevant.

A comparison of the evaluations by journalists of the Sydney J2J sessions with our own evaluations of the same sessions revealed concordant excellent grades for five of seven lectures, while the other two were only discordant between good vs. excellent grades. This is consistent with the observation that the presentations were of high caliber in regard to the objective of educating journalists.

### 4. Evaluations of News Reports

**Figure 1:**
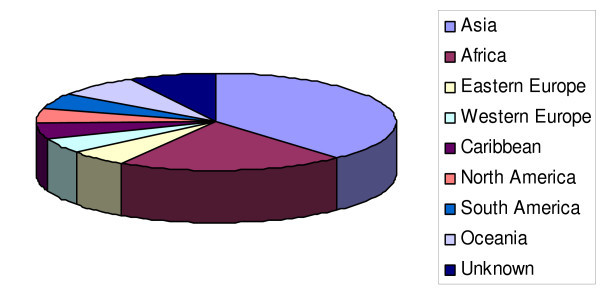
The reports from journalists who participated in the J2J program and filed HIV/AIDS primarily represented areas of the world where HIV/AIDS is, or will likely be, of great impact.

Thirty-nine news reports from those that were written in English or had an accompanying English translation were randomly chosen for review by two evaluators. In almost all cases, the reports were from journalists working on developing countries (Figure 1).

Those in the categories of excellent and good were grouped together and the extent of agreement between the evaluations was determined. Discordant evaluations were adjudicated by an additional reviewer, if necessary. Reports in English were deliberately overrepresented in the sample analyzed, as the reviewers were mainly English-speaking. In almost all cases, the reports were from journalists working in developing countries (see Figure 1).

In regard to quality of the reports (relevance and accuracy), thirty-three of the 39 (84%) reports from all of the J2J sessions evaluated were found to be good or excellent. The topics discussed in these journalists' reports are summarized in Table 2.

**Table 2:** Topics discussed in journalists reports

Topics	Number of reports
Global epidemiology and public health priorities of HIV/AIDS	8
Conference coverage	6
Innovative methods to increase public awareness about HIV/AIDS in developing countries	2
Coverage of government responses	1
Restricted ART access in developing country settings	5
Information on low use of MTCT prevention, pediatric ARV limitations, and the growing problems of orphans due to HIV/AIDS worldwide	3
Culturally-related responses to prevention strategies, importance of youth, women, and NGOs in fighting HIV/AIDS	2
Coverage of J2J the program and its benefits	2
Coverage of people with HIV/AIDS, stigma-related issues and family effects of MTCT of HIV/AIDS	2
Discussion on social aspects of HIV transmission in heavily-affected areas, risk reduction strategies in high-risk populations, enhancing prevention strategies, non-typical higher risk populations.	5
Information about microbicide trials and ARV treatment in case of rape.	1
Financial support for HIV/AIDS-affected people in developing countries	1

Some reports covered more than one topic

### 5. Online Survey

We emailed a request to complete an online survey to 160 journalists. Seventeen e-mail messages did not reach recipients. Forty-two journalists completed the survey.

**Figure 2:**
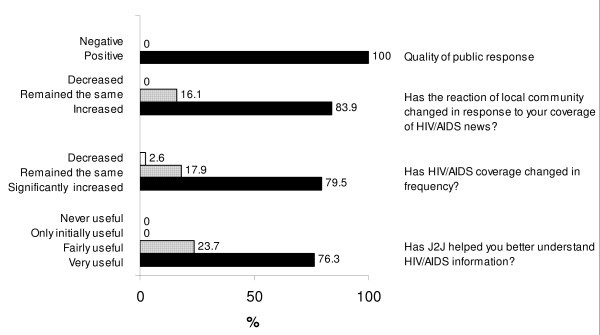
This figure presents the responses given by J2J attendees to questions about their perception on usefulness of the J2J program. The data was obtained through an online survey.

The respondents were almost unanimous in judging that the J2J program was very useful and 79% of them have increased their reporting of HIV/AIDS since the conference (Figure 2).

In addition, the knowledge gained has continued to help journalists in their subsequent coverage of HIV/AIDS. No journalist had a negative attitude toward either the J2J program or community groups working in the HIV/AIDS field. The great majority of journalists perceived that their coverage of the IAS conferences was greeted more enthusiastically by the communities that they serve than would have been the case if not for the J2J program.

#### Print and Radio Journalists

The majority of the J2J journalist fellows wrote newspaper articles or reports to be posted on the worldwide web. The median number of print articles and radio presentations by journalists has been 3 and 4, respectively, per month in the time since the conference. Radio and newspaper coverage are the most likely means for dissemination of information in the developing world, since only minimal infrastructure is required.

#### Television

Television was used less frequently as a medium by journalists in the developing world, although 6 of the J2J journalists aired HIV/AIDS related programs on TV. The broadcasting frequency of each report varied. One was aired once, whilst another aired four times in one week. One journalist reported that his/her program was broadcast monthly.

### 6. Examples of Experiences of Journalists

Two sources of descriptive evaluations of the program were available. A database from the J2J Sydney program and an additional survey carried out by our team. Of note, the vast majority of comments from the journalist evaluation database was favorable and acknowledged appropriate organization, pertinence of the program, and usefulness of the presentations.

The following comments provide a reasonable idea of some journalists' impressions of the J2J program:

"Honestly, without the J2J training, I would have spent half of my time at the IAS conference referring to either a science dictionary or googling up certain complicated scientific phrases."

"The AIDS Denialist session was fantastic: it's good to be reminded of tactics for handling the denialists, which are a real headache here in South Africa."

"In addition to its comprehensiveness, the programme represented a huge effort to reach out to and include journalists from the developing world."

"To me it was the best effort. But I would suggest if every one of us can share his/her stories done afterwards because it would help every one of us."

"It is good to have a hands-on training on science reporting for the AIDS pathogenesis, treatment and prevention conference"

"I think what was on offer at this years training program was perfect. If you can inspire someone to act and feel different about how they view HIV/AIDS in just a week then you have succeeded. Please know your program is inspirational."

"I feel the program offered a thorough overview of the HIV/AIDS pandemic and gave me a unique opportunity to share observations and ideas with colleagues from around the world."

There were few comments on program failures.

"Next time, NPF could improve its trainings by advising or asking presenters to avoid scientific jargon., That is, putting their presentation in simpler words that could be understood by ordinary people including journalists. And they should be brief and to the point."

"I would suggest that next time the training should be 5 days long and we should have more field visits to have a face on what we would be doing. Thanks for taking us to Kirketon Xentre. We really learnt a lot and we have since adopted their approach here in..."

## Discussion

### Strengths of the program

The program appears to have fully met its main purpose of enabling journalists to effectively transmit medical, epidemiological and scientific information to the general public in lay language.

This, in turn, may to help to raise the interest and awareness of the general public in developing countries about resources that can effectively be mobilized to both reduce transmission of HIV and to treat those living with HIV/AIDS. This is important since the vast majority of journalists trained by the J2J program come from and work in developing countries in which HIV/AIDS is a major public health threat.

### Weaknesses of the program and opportunities for improvement

Although the program does an excellent job at enhancing journalistic skills to translate scientific information into lay language, there appears to be a shortage of information as to what journalists should be doing at a local level. Should they be querying their own local communities with respect to local practices and the role of local health promotion authorities? This subject is complex, and, in some countries, it should be recognized that journalists may sometimes feel intimidated by the types of questions they might wish to ask.

Second, several scientific presentations within the J2J program did not attempt to use non-scientific terminology and/or the presenters did not take the time to try to explain their findings to journalists in lay language. Emphasis needs to be placed on the transmission of scientific concepts over a range of HIV/AIDS disciplines.

Third, a weak representation of the private sector was evident in all the J2J programs. Clearly, journalists would like to have the opportunity to ask questions to representatives of the pharmaceutical industry (including generic industry spokespersons). This is a key area for consideration, since the public is poorly informed in general about the roles played by drug companies in scientific research and may be easily seduced by 'conspiracy theories' that attribute false motives to companies. Responsible reporting on relationships between the private and public sectors, including academia, may help to quench misconceptions.

Finally, presentations of exemplary work by leading world-class HIV/AIDS journalists might also enhance the J2J curriculum. Less experienced journalists might be paired with more experienced 'mentor' journalists from their own countries, as well as from developed countries, for in depth discussions. Former fellows might also be able to share experiences with new fellows and help the latter to improve their communications skills. There could then be a 'trickle-down effect' if journalists were to conduct smaller, albeit less ambitious J2J-like programs, in their own countries.

### Implications for global public health

The need for education of communities about HIV is evident. Several reports have documented insufficient knowledge in populations at risk of acquiring HIV infection [8-10]. In this regard, the mass media could have a positive impact on improving the public's knowledge of HIV. For instance, media are able to affect audience behavior in a way that might favor prevention (e.g. discussion of HIV/AIDS with a partner, awareness that consistent condom use reduces HIV risk, asking about condom use at last intercourse, or increasing voluntary HIV testing) [4,11-13].

The World Health Organization has stated that impact may vary, depending on the place and campaign, but that comprehensive mass media programs are valuable in helping to change HIV/AIDS-related behavior, at least among young people in developing countries [1]. Therefore, education of journalists, who are often partners in such efforts worldwide, is consistent with the types of activities that advance public health.

The J2J program has an opportunity to engage in outreach to help direct and/or support international education campaigns through the networks that have now been established. A continuous and synchronized effort to promote education of communities through written publications and/or radio programs might be established using the broad human resource represented by the J2J program. The creation of material based on the J2J presentations and local replication of similar programs could be encouraged, and could also be carried out in languages other than English. Ongoing feedback from such efforts could then be used to improve the overall effort, which could be implemented and locally tailored to regional needs for use in subsequent initiatives.

## Limitations of the study

The response rate for the survey was only 26%, evidently raising the issue of bias. On the other hand, favorable grades were given to the j2j program by attendees who evaluated the program at previous conferences and these were consistent with the later grading found through the survey.

Despite the heterogeneous educational background of the journalists attending the J2J program, we observed a high quality of accuracy and pertinence in the reports written by attendees. Altogether, these observations suggest a beneficial effect of the program on the communication skills of the journalists in the HIV/AIDS field. Nevertheless, a sample of reports by the journalists before the J2J session would have been ideal for comparison with those available after the session. Unfortunately, such information was not available to us.

For future evaluations, and in order to accurately determine the effect of J2J on journalists' skills, it might be advisable to obtain and evaluate a baseline set of reports from the invited journalists before the session.

Although we cannot definitively conclude that J2J improved skills in reporting of HIV/AIDS in general, the perception from attendees at the end of the each J2J program and those who responded to our survey were all positive suggesting that the goals of the program were realized.

Usually, lack of response to a survey represents low motivation to spend time answering questions and not necessarily a negative perception of the issue involved. In addition, emails to contact journalists in developing countries might not be the best strategy for future surveys since internet access may be limited or unstable for a proportion of potential respondents.

## Conclusion

The J2J program of the National Press Foundation has accomplished its main goal of gathering journalists from around the world to be trained in how to better report HIV/AIDS news.

Journalists have consistently indicated that the program is highly useful and that it enables them to cover and inform the public in a variety of areas: experiences of people living with HIV/AIDS, impact on society, the reasons for stigma, how to work toward destigmatization of HIV, hopes and limitations of current therapy including issues of drug access in developing countries, prospects for novel therapeutic and prevention initiatives, and the successes and failures of research and/or public health measures.

Vital information in each of these areas needs to reach the general public, who will ultimately decide what it is important to pay attention to and in which areas to establish priorities.

Journalist reports are an effective means of providing information on HIV awareness to vulnerable populations, hopefully helping to lower rates of infection and educating those who are infected by HIV to seek adequate help. Public awareness can help to guide public opinion and influence government policy in a positive way and to counter stigma, which is often a result of misperceptions about HIV/AIDS. Journalists play important roles in each of these areas and the J2J program has been key in educating journalists worldwide to do their jobs better.

## Competing interests

MAW was an invited speaker at the J2J Conference in Sydney.

## Authors' contributions

JLMC led in the study design, data analysis and manuscript preparation. CFI participated in the study design, data analysis and manuscript preparation. MN and SMS participated in review and evaluation of news reports and of the J2J program's curriculum. MAW participated in study design, evaluation of news reports, and manuscript preparation.

## Supplementary Material

Additional file 1 Survey Questionnaire. The questionnaire used for the on-line survey. Click here for file
