# Supplementary material for: Benefits of an educational program for journalists on media coverage of HIV/AIDS in developing countries
Source: J Int AIDS Soc. 2008 Sep 22;11:2. doi: 10.1186/1758-2652-11-2 (PMC2580037; doi:10.1186/1758-2652-11-2)
Supplement: Additional file 1 — Survey Questionnaire. The questionnaire used for the on-line survey. [file 1758-2652-11-2-S1.doc]

**Additional file 1. Survey Questionnaire**

1. **Do you think that what you learned during the National Press Foundation Journalist Training Program on HIV/AIDS is still helping you to better understand scientific information on the topic of HIV/AIDS?**
   1. It is still very useful
   2. It is still fairly useful
   3. It was initially useful but now not
   4. It has never been useful
2. **Has your coverage of HIV/AIDS-related news changed as a result of the training program?**
   1. It has significantly increased
   2. It has remained the same
   3. It has decreased
3. **How often are you currently covering or participating in activities related to HIV/AIDS news. WRITTEN MEDIA (magazines, journals or websites). Please enter a number in ONLY ONE of the following choices:**
   1. Number of articles published weekly
   2. Number of articles published monthly
   3. Number of articles published yearly
   4. I am not involved in written media (IF THIS APPLIES, PLEASE TYPE THE NUMBER 1)
4. **How often are you currently covering or participating in activities related to HIV/AIDS news. RADIO. Please enter a number in ONLY ONE of the following choices:**
   1. Number of programs aired weekly
   2. Number of programs aired monthly
   3. Number of programs aired yearly
   4. I am not involved in radio (IF THIS APPLIES, PLEASE TYPE 1 THE NUMBER 1)
5. **How often are you currently covering or participating in activities related to HIV/AIDS news. TELEVISION. Please enter a number in ONLY ONE of the following choices:**
   1. Number of programs aired weekly
   2. Number of programs aired monthly
   3. Number of programs aired yearly
   4. I am not involved in television (IF THIS APPLIES, PLEASE TYPE 1)
6. **Has the reaction of the community (opinion letters, phone calls, visits) in your area of influence changed in response to your reports on HIV/AIDS? The frequency of public response has**
   1. Increased
   2. Remained the same
   3. Decreased
7. **Has the reaction of the community in your area of influence changed in response to your reports on HIV/AIDS? Regarding the quality of public response, is the feedback from your community mainly**
   1. positive
   2. negative
8. **Please provide your e-mail address**
9. **Please, briefly provide us with one example of a report/program that you consider as a success and one that you consider as a failure. You are encouraged to e-mail us text or pdf files or scanned documents (just reply to our e-mail address and attach files).**
